# Evaluation of *Cordyceps sinensis* Quality in 15 Production Areas Using Metabolomics and the Membership Function Method

**DOI:** 10.3390/jof10050356

**Published:** 2024-05-16

**Authors:** Tao Wang, Chuyu Tang, Hui He, Zhengfei Cao, Mengjun Xiao, Min He, Jianzhao Qi, Yuling Li, Xiuzhang Li

**Affiliations:** 1 State Key Laboratory of Plateau Ecology and Agriculture, Qinghai Academy of Animal Science and Veterinary Medicine, Qinghai University, Xining 810016, China; 13085500761@163.com (T.W.); chuyutang0410@163.com (C.T.); he15226330573@163.com (H.H.); c1474477969@163.com (Z.C.); 15574237597@163.com (M.X.); himi1228@163.com (M.H.); 2 College of Chemistry and Pharmacy, Northwest A&F University, Xianyang 712100, China; qjz@nwafu.edu.cn; 3 State Key Laboratory of Plateau Ecology and Agriculture, Qinghai Academy of Animal and Veterinary Science, Xining 810016, China

**Keywords:** *Cordyceps sinensis*, metabolomics, biomarkers, quality evaluation

## Abstract

*Cordyceps sinensis* is a precious medicinal and edible fungus, which is widely used in body health care and disease prevention. The current research focuses on the comparison of metabolite characteristics between a small number of samples and lacks a comprehensive evaluation of the quality of *C. sinensis* in a large-scale space. In this study, LC-MS/MS, principal component analysis (PCA), hierarchical cluster analysis (HCA), and the membership function method were used to comprehensively evaluate the characteristics and quality of metabolites in 15 main producing areas of *C. sinensis* in China. The results showed that a total of 130 categories, 14 supercategories, and 1718 metabolites were identified. Carboxylic acids and derivatives, fatty acyls, organo-oxygen compounds, benzene and substituted derivatives, prenol lipids, and glycerophospholipids were the main components of *C. sinensis*. The HCA analysis and KEGG pathway enrichment analysis of 559 differentially accumulated metabolites (DAMs) showed that the accumulation models of fatty acids and conjugates and carbohydrates and carbohydrate conjugates in glycerophospholipid metabolism and arginine and proline metabolism may be one of the reasons for the quality differences in *C. sinensis* in different producing areas. In addition, a total of 18 biomarkers were identified and validated, which had a significant discrimination effect on the samples (*p* < 0.05). Overall, YS, BR, and ZD, with the highest membership function values, are rich and balanced in nutrients. They are excellent raw materials for the development of functional foods and provide scientific guidance for consumers to nourish health care.

## 1. Introduction

*Cordyceps sinensis* is an insect–fungal complex formed by *Ophiocordyceps sinensis* after infecting the larvae of the bat moth and causing the death of the host. It is a precious biological resource [1]. As a famous Chinese herbal medicine widely used in medical treatment, it has a history of more than 300 years [2]. In the world, the species of *C. sinensis* are mainly distributed in North America, East Asia, and Southeast Asian countries, and most species are from the Qinghai–Tibet Plateau and its surrounding areas in China [3]. *C. sinensis* is distributed in a stepwise manner, with the increase in altitude in China. It has not been or has rarely been in the low-altitude area (3500 m). The main producing areas are Qinghai and Tibet [4]. The great altitude difference leads to different growth environments of *C. sinensis* in different producing areas [5], which is finally manifested as the difference in its metabolic spectrum.

*C. sinensis* is rich in a variety of bioactive components, such as amino acids, sphingolipids, adenosine, carbohydrates, polysaccharides, mannitol, and nucleosides, which exhibit different medicinal functions [6,7,8]. The in vitro antioxidant capacity test of *C. sinensis* water extract showed that it had a strong ability to scavenge hydroxyl radicals and DPPH radicals and also had a strong reducing ability, which was attributed to its rich variety in amino acids, among which glutamic acid and arginine were excellent [9]. Nucleoside compounds are considered to be involved in the treatment of cancer. Mannitol can inhibit the formation of superoxide anions and the release of elastase, and it shows strong cytotoxicity towards cancer cells A549, PANC-1, and McF-7 [10,11]. When polysaccharides were used as additives (200 μM) to feed nematodes, researchers found that the average lifespan of the nematodes was prolonged by 11.3% [12]. This ingredient is rich in a variety of proteins that have been reported to have multiple biological effects, including anti-fungal and anti-virus properties and a role in regulating immune function [13]. Therefore, *C. sinensis* is also widely regarded by nutritionists and professional health care workers. The tannin and dihydromyricetin extracted from the aqueous extract of *C. sinensis* exhibited strong 1,1-Diphenyl-2-picrylhydrazyl radical 2,2-Diphenyl-1-(2,4,6-trinitrophenyl)hydrazyl and 2,2′-azino-bis(3-ethylbenzothiazoline-6-sulfonic acid) (ABTS) radical scavenging activities and reducing power [14]. Encouragingly, the hot-water extract of *C. sinensis* can promote the secretion of paclitaxel and significantly inhibit taxol-induced cytotoxicity in mice [15]. Finally, *C. sinensis* showed specific anti-fatigue effects in clinical trials of sub-health status and the treatment of fatigue status [16].

In the past 20 years, *C. sinensis*, as a good functional food and drug development raw material, has been the subject of published studies, with a lot of good comments, but its edible value and medicinal value depend on its origin [17]. Different sources of *C. sinensis* have different sensory qualities, metabolite compositions, and flavors [18]. In particular, *C. sinensis* in high-altitude areas such as the Yushu Tibetan Autonomous Prefecture, the Qinghai province, Naqu city, and the Tibet Autonomous Region (TAR) is rich in metabolite species [19,20]. These factors make the comprehensive evaluation of the quality of *C. sinensis* in many producing areas around the Qinghai–Tibet Plateau one of the current academic hotspots. In this study, LC-MS/MS liquid chromatography–tandem mass spectrometry was used to qualitatively and quantitatively analyze the metabolites of *C. sinensis* from 15 producing areas. According to the screened and identified biomarkers, the comprehensive evaluation of the quality of *C. sinensis* from 15 producing areas was completed, which provided a scientific basis for its edible and medicinal use.

## 2. Materials and Methods

### 2.1. Sample and Data Collection

**Figure 1:**
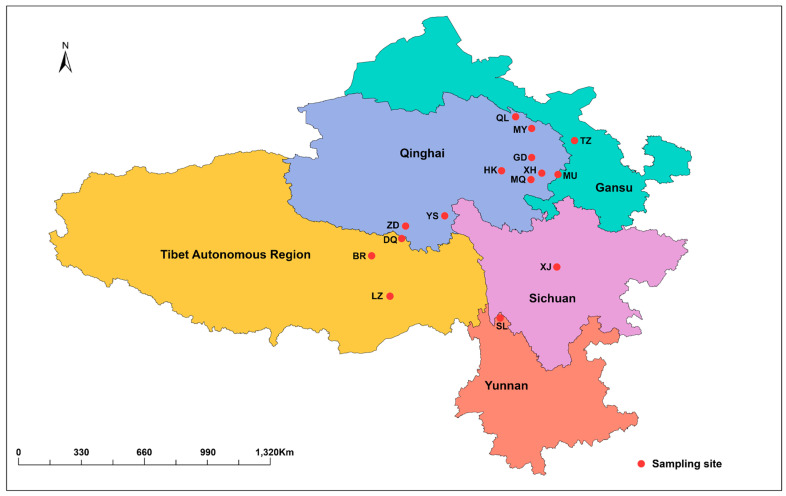
Sample distribution of *C. sinensis* from 15 producing areas. BR is Biru County in the TAR; DQ is Dingqing County in the TAR; LZ is Linzhi County in the TAR; ZD is Zaduo County in the Qinghai province; YS is the Yushu Tibetan Autonomous Prefecture in the Qinghai province; HK is Xinghai Country in the Qinghai province; MQ is Maqin County in the Qinghai province; XH is Xunhua County in the Qinghai province; GD is Guide County in the Qinghai province; MY is Menyuan County in the Qinghai province; QL is Qilian County in the Qinghai province; TZ is Tianzhu County in the Gansu province; MQ is Maqu County in the Gansu province; XJ is Xiaojin County in the Sichuan province; and SL is Shangri-La city in the Yunnan province.

From the 7 May 2023 to the 11 June 2023, the research group purchased *C. sinensis* from 15 producing areas (biological repeats = 6) in the Qinghai province, Sichuan province, Yunnan province, Gansu province, and TAR in China as experimental materials to study the nutritional components of *C. sinensis* in different producing areas (Figure 1). Samples of *C. sinensis* at the same maturity stage were selected from each producing area, and 6 biological replicates were set. This study does not involve special plant or animal reserves nor does it require special permits. All the collected materials were frozen at −80 °C. We downloaded climate data (https://data.cma.cn/, accessed on 16 November 2023), collated as background data (Table S1), from the National Meteorological Science Data Center of China Climate Science Data Center.

### 2.2. Sample Pretreatment and Metabolite Extraction

The stroma and sclerotia of *C. sinensis* were rinsed with distilled water three times, then 100 mg of the sample was weighed into a 2 mL centrifuge tube, and a grinding bead with a diameter of 6 mm was added to assist with grinding. A total of 100 mg samples was added to 400 μL extractant (80% methanol + 20% water) and ground for 6 min (−10 °C, 50 Hz). After low-temperature extraction for 30 min at 5 °C and 40 KHz, the samples were centrifuged at 4 °C and 13,000 rpm for 15 min. Then, the supernatant was weighed and injected into an injection bottle with a 0.22 um filter membrane for detection. A total of 10 μL of the extract of all the samples was taken to prepare a quality control (QC) sample to investigate the stability of the entire detection process. The reagents used in this study were all analytically pure purchased from Merck S.A. (Merch, Beijing, China).

### 2.3. LC-MS/MS

The extraction and detection of the metabolites were based on the existing research results of the *Cordyceps sinensis* Research Laboratory and modified the reported references [21]. The samples were detected by the ultra-high-performance liquid chromatography–tandem Fourier-transform mass spectrometry UHPLC-Q Exactive HF-X system. The chromatographic conditions were as follows: chromatographic column ACQUITY UPLC HSS T3 (100 mm × 2.1 mm i.d., 1.8 μm; Waters, Milford, CT, USA); the mobile phase A was 95% water + 5% acetonitrile (containing 0.1% formic acid); and the mobile phase B was 47.5% acetonitrile + 47.5% isopropanol + 5% water (containing 0.1% formic acid). The injection volume was 3 μL, and the column temperature was 40 °C. The samples were ionized by electrospray ionization, and the mass spectrometry signals were collected by ESI+ and ESI-scanning modes, respectively. The mass spectrometry conditions were the following: scan type (70–1050 m/z); sheath gas flow rate (50 arb); aux gas flow rate (13 arb); heater temp (425 °C); capillary temp (325 °C); spray voltage (±3500 V); s-Lens RF level (50); resolution (Full MS60,000); and resolution (7500 MS2). 

### 2.4. Metabolite Identification and Quantitative Analysis

Both positive (POS)- and negative (NEG)-mode data were used in this study. The measured raw data were imported into Progenesis QI (Waters Corporation, Milford, CT, USA) for line filtering, peak identification, integral, retention time correction, and peak pair. Finally, the data matrix of the retention time, mass-to-charge ratio, and peak intensity was obtained. The MS and MS/MS mass spectrometry information was matched with the metabolic database (HMDB, Metlin and KEGG). The MS mass error was set to be less than 10 ppm, and the metabolites were identified according to the secondary mass spectrometry matching annotation. To facilitate the subsequent data analysis, the data matrix was normalized, and a log transformation was performed to correct the heteroscedasticity of the dataset, reduce the asymmetry in the data structure, and improve the normality of the data. 

### 2.5. Acquisition of the Integrated Membership Function

The integrated membership function values of 15 samples from different producing areas were calculated using the 18 verified biochemical markers, which were calculated with the following equation [22,23]:

$$X_{ab}^{+}\left(n\right)=\left(X_{ab}-X_{bmin}\right)/\left(X_{bmax}-X_{bmin}\right)\;X_{ab}^{-}\left(n\right)=1-X_{ab}^{+}\left(n\right)$$

Among them, $X_{ab}^{+}\left(n\right)$ represents the integrated membership function value that is positively correlated with the sample, $X_{ab}^{-}\left(n\right)$ represents the integrated membership function value that is negatively correlated with the sample (Table S2), $X_{ab}$ is the mean value of the measured value of the *b* index of the sample, $X_{bmin}$ is the minimum value of the *b* index of all the samples, and $X_{bmax}$ is the maximum value opposite to it.

## 3. Results

### 3.1. Overview of C. sinensis Metabolites

**Figure 2:**
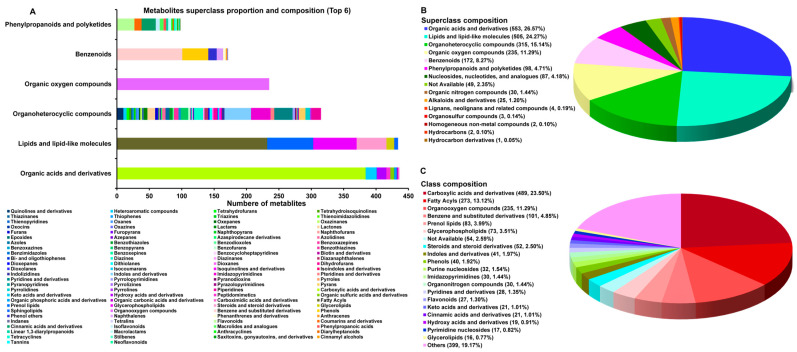
Composition of metabolites of *C. sinensis* from 15 producing areas: (**A**) the percentage histogram of the top 6 superclasses; (**B**) the pie chart of the superclasses; and (**C**) the pie chart of the classes.

QC samples were obtained by merging all the samples to test the stability and reliability of the system. In the obtained total ion current chromatogram, it was observed that the QC control sample ran stably before 8 min, and the retention time and peak intensity of POS and NEG had good repeatability, indicating that data determination was reliable (Figure S1A). The relative standard deviation of POS and NEG was less than 30%, and the proportion of peak accumulation was more than 70%, indicating that the test method and determination data were reliable (Figure S1B). A total of 1826 metabolites (Table S2) were detected in this study, and 1718 metabolites (POS: 800 metabolites; NEG: 918 metabolites) could be identified. They were divided into 14 superclasses, 130 classes, and 266 subclasses (Table S3) (Figure 2A). *C. sinensis* was rich in organic acids and derivatives (553 metabolites, 26.57%), lipids and lipid-like molecules (505 metabolites, 24.27%), organoheterocyclic compounds (315 metabolites, 15.14%), organic oxygen compounds (235 metabolites,11.29%), benzenoids (172 metabolites, 8.27%), and phenylpropanoids and polyketides (98 metabolites, 4.71%) (Figure 2B). It was rich in carboxylic acids and derivatives (489 metabolites, 23.50%), fatty acyls (273 metabolites, 13.12%), organo-oxygen compounds (235 metabolites, 11.29%), benzene and substituted derivatives (101 metabolites, 4.85%), prenol lipids (83 metabolites, 3.99%), and glycerophospholipids (73 metabolites, 3.51%) (Figure 2C). 

The carboxylic acids and derivatives (88.43%) were the main metabolites of organic acids and derivatives, and the content of fatty acyls (54.06%) in the lipids and lipid-like molecules was the highest. The benzenoids had the highest content of benzene and substituted derivatives (58.72%). The organoheterocyclic compounds had the most class diversity (64 classes), while the organic oxygen compounds were all composed of organo-oxygen compounds. The composition of the various metabolites in the phenylpropanoids and polyketides was similar. These conclusions provided valuable data for the medicinal value of *C. sinensis* and the mining of potential biomarkers. 

### 3.2. Multivariate Statistical Analysis of Metabolites

**Figure 3:**
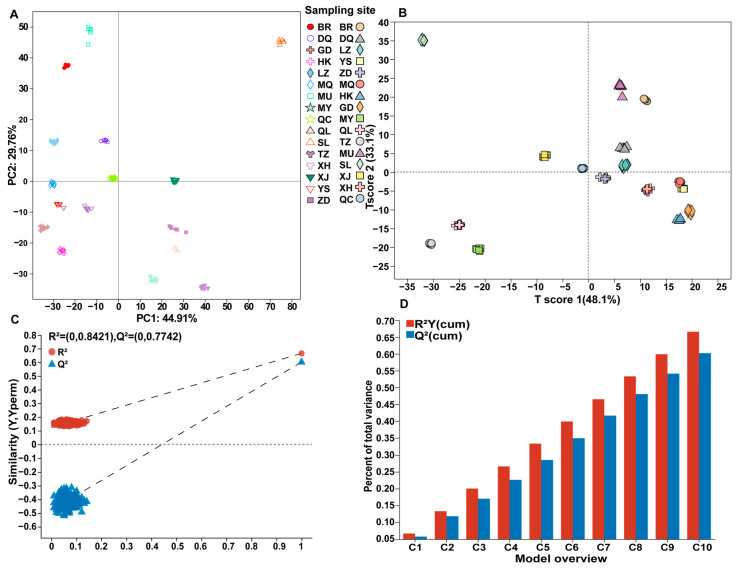
Multivariate statistical analysis of metabolites of *C. sinensis* from 15 producing areas: (**A**) PCA analysis score map; (**B**) PLS-DA model score map; (**C**) PLS-DA model validation map; and (**D**) PLS-DA principal component number selection map.

**Figure 4:**
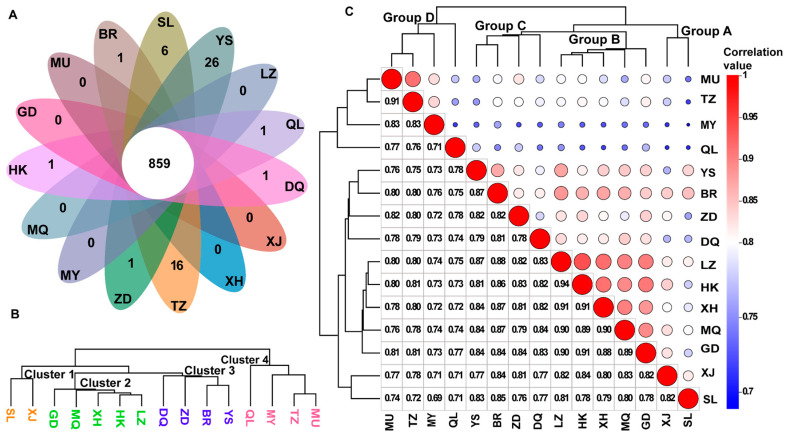
The relationship among the samples of *C. sinensis* from 15 producing areas: (**A**) Venn diagram; (**B**) sample-clustering diagram; and (**C**) sample correlation diagram.

Multivariate statistical analysis was used to analyze the differences in metabolites between *C. sinensis* from 15 producing areas. The results of the PCA showed that the first two principal components accounted for 74.67% (PC1 = 44.91%; PC2 = 29.76%), indicating that the metabolites could effectively distinguish the samples. The distribution of the samples was highly dispersed, indicating that the metabolites of *C. sinensis* from different habitats were quite different (Figure 3A). To accurately find the DAMs between the samples, the partial least-squares discriminant analysis (PLS-DA) model was used in this study, and all the samples were distinguished without overlapping (Figure 3B). A permutation test of 200 times was used to test the validity and fitting effect of the model. The results showed that $R_{Y}^{2}$ = 0.84 and Q^2^ = 0.77, and the model had good fitting effect and predictability (Figure 3C,D). According to the types of metabolites, Venn analysis was performed on the samples, and it was observed that samples YS and TZ had the most metabolite diversity (Figure 4A). The abundance of metabolites was used for sample clustering and a correlation analysis. The results showed that *C. sinensis* specimens from 15 producing areas were divided into four categories. Group A was XJ and SL; group B included GD, MQ, XH, HK, and LZ; group C included ZD, DQ, BR, and YS; and group D included MU, TZ, MY, and QL (Figure 4B,C). The similarity between the three groups of samples A, B, and C was higher, showing the distribution pattern in and around the Qinghai–Tibet Plateau.

### 3.3. Identification of DAMs and Enrichment Pathway Analysis

**Figure 5:**
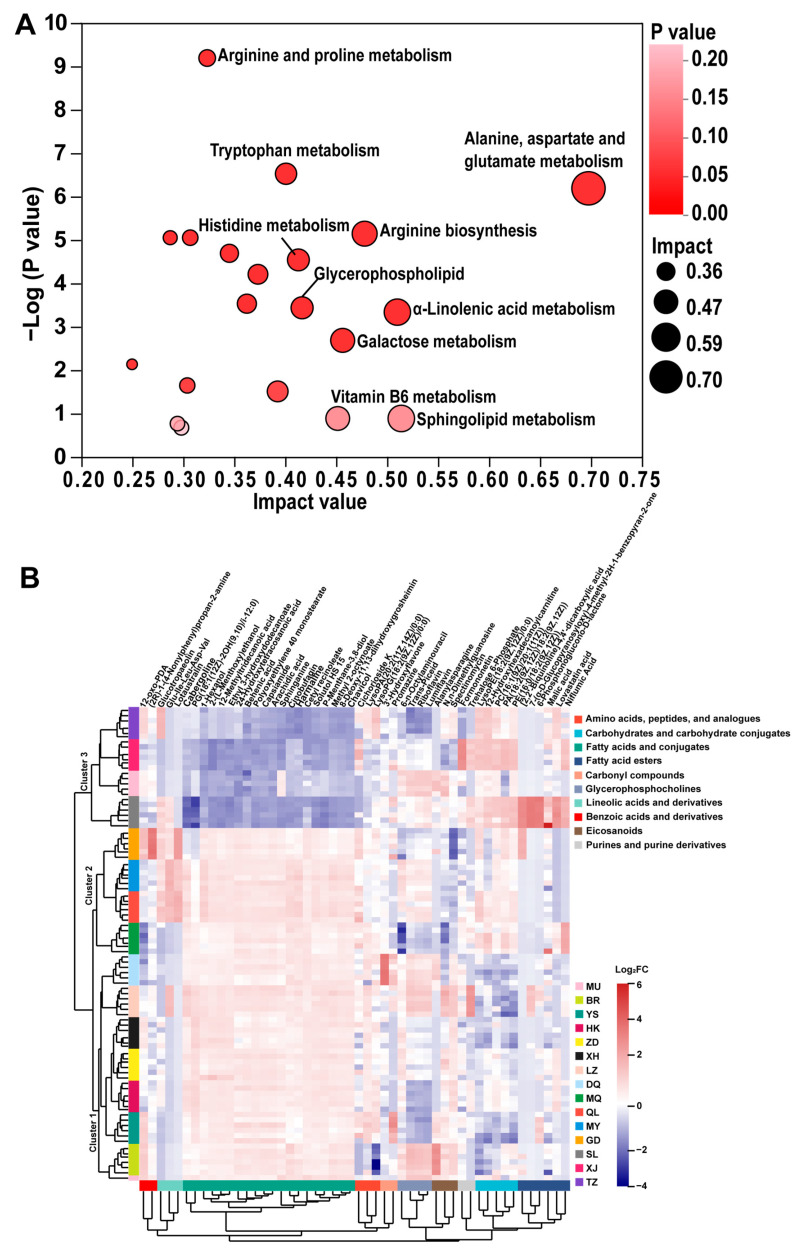
Comprehensive analysis of DAMs: (**A**) enrichment analysis of KEGG topology analysis of DAMs in *C. sinensis* from 15 producing areas; and (**B**) hierarchical clustering analysis of 50 DAMs before the VIP value of *C. sinensis* from 15 producing areas.

Based on the score of each metabolite in the PLS-DA model, its variable importance in projection (VIP) value was calculated. Differentially accumulated metabolites (DAMs) were screened by VIP ≥ 2 and FDR < 0.5, and a KEGG metabolic pathway enrichment analysis (Table S4) was performed. A total of 559 metabolites were annotated into 76 metabolic pathways, the top 20 of which are visualized by KEGG in Figure 5A. The alanine, aspartate, and glutamate metabolism, the sphingolipid metabolism, the α-Linolenic acid metabolism, and arginine biosynthesis were significant enrichment pathways for DAMs (Figure 5A).

The HCA of the top 50 DAMs showed that the samples clustered into the same class showed similar metabolite compositions, and the metabolites clustered into the same class showed similar or complementary physiological functions. This study found that the samples were clustered into three categories: cluster 1 included BR, YS, HK, ZD, XH, LZ, and DQ; cluster 2 included MQ, QL, MY, and GD; and cluster 3 included SL, MU, XJ, and TZ (Figure 5B). Among them, the relationship between cluster 1 and cluster 2 was closer, which indicated that the metabolic spectrum characteristics of the samples in cluster 1 and cluster 2 were more similar. The 15 samples were also divided into two categories: BR, YS, HK, ZD, XH, LZ, DQ, MQ, QL, MY, and GD were located in the Qinghai–Tibet Plateau, including the Qinghai province and the TAR; SL, MU, XJ, and TZ were around the Qinghai–Tibet Plateau, including the Gansu province, the Sichuan province, and the Yunnan province.

The top 50 DAMs were clustered into 10 categories, with the most abundant being fatty acids and conjugates (20 metabolites, 40%), followed by fatty acid esters (6 metabolites, 12%), carbohydrates and carbohydrate conjugates (5 metabolites, 10%), and glycerophosphocholines (4 metabolites, 8%). Among them, *C. sinensis* specimens belonging to the Qinghai–Tibet Plateau were mostly rich in fatty acids, their conjugates, and glycerophosphocholines; most of the *C. sinensis* samples produced around the Qinghai–Tibet Plateau were rich in fatty acid esters, carbohydrates, and carbohydrate conjugates. Our study also showed that these four types of substances were the most different metabolites of *C. sinensis* in the 15 producing areas and potential biomarkers. In addition, fatty acids and conjugates could qualitatively distinguish all the samples, and the identification effect of other DAMs on the samples cannot be observed from the figure above. Therefore, a quantitative analysis of the 50 DAMs is still needed to determine their identification effect on the samples.

### 3.4. Validation of Biomarkers

**Figure 6:**
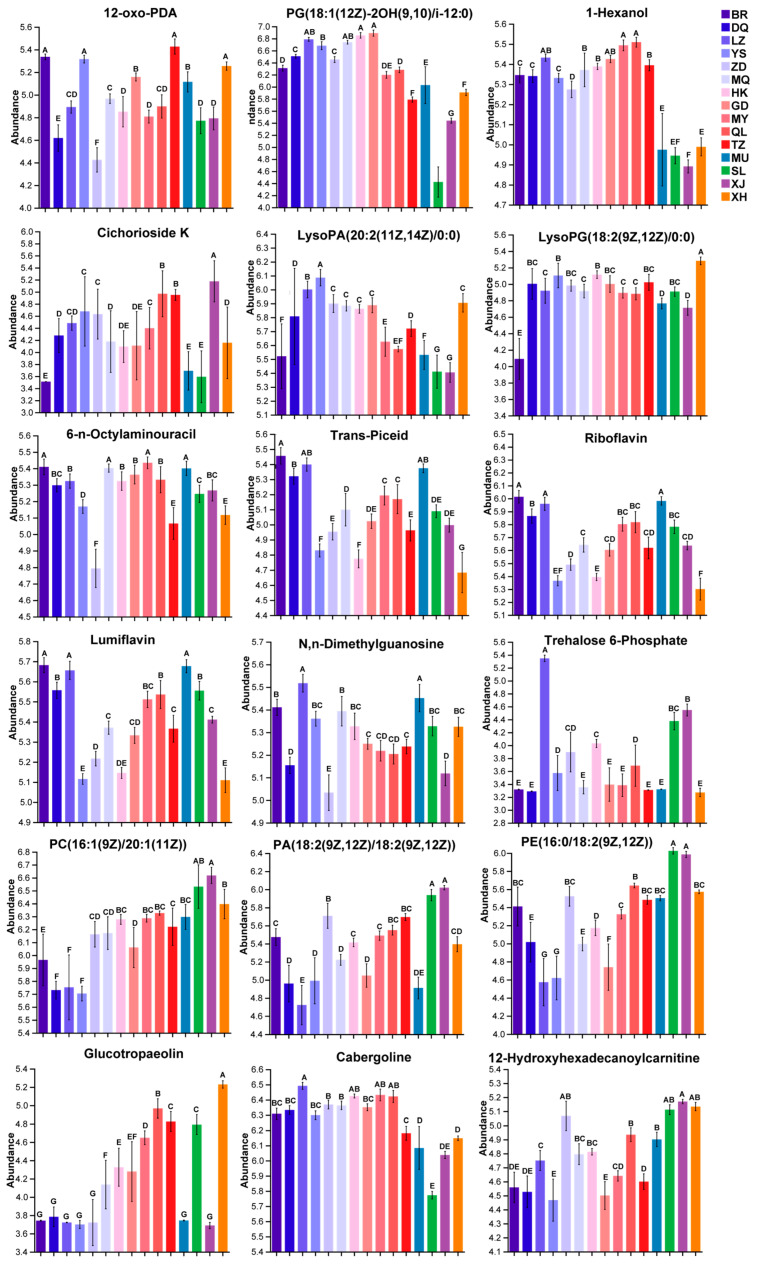
Single-factor analysis of the 18 DAMs. The uppercase letters in the figure indicate that the abundance of metabolites is significantly different at the level of *p* < 0.05.

To determine whether these DAMs could be used as effective biomarkers and verify their identification effect on the samples, the abundance of these DAMs in *C. sinensis* from 15 producing areas was analyzed by a single-factor analysis. In this study, the following DAMs showed excellent performance in the identification of the samples (Figure S2): 12-oxo-PDA, 1-Hexanol, cichorioside K, lysopa(20:2(11z,14z)/0:0), lysopg(18:2(9z,12z)/0:0), 6-n-octylaminouracil, trans-piceid, riboflavin, lumiflavin, n, n-dimethylguanonine, trehalose 6-phosphate, pc(16:1(9z)/20:1(11z)), pa(18:2(9z,12z)/18:2(9z,12z)), pe(16:0/18:2(9z,12z)), glucotropaeolin, cabergoline, pg(18:1(12z)-2oh(9,10)/i-12:0), and 12-hydroxyhexadecanoylcarnitine (Figure 6). The abundance of these 18 metabolites was significantly different among the samples (*p* < 0.05), and they were excellent biomarkers for the *C. sinensis* samples from 15 different regions. This provides a scientific basis for the quality evaluation, grade identification, and traceability analysis of *C. sinensis*.

### 3.5. Comprehensive Quality Evaluation of C. sinensis from 15 Producing Areas Based on Biomarkers

**Figure 7:**
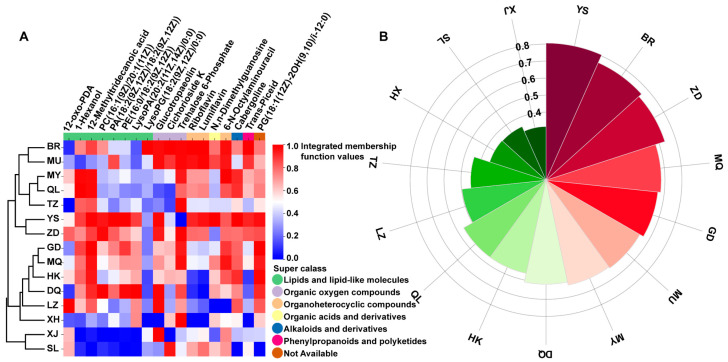
Comprehensive analysis of *C. sinensis* quality in 15 producing areas: (**A**) radar map of membership scores for 18 biomarkers from 15 producing areas; and (**B**) average radar chart of integrated membership function of *C. sinensis* in 15 producing areas.

To further measure the quality of *C. sinensis* in 15 producing areas, it was comprehensively evaluated by 18 biomarkers screened by the membership function method (Table S6). The results showed that the composition of these biomarkers in *C. sinensis* samples from 15 habitats was quite different, indicating that these *C. sinensis* specimens may have different medicinal functions and health care values (Figure 7A). For example, because YS, ZD, and DQ were rich in 12-methyltridecanoic acid, 12-oxo-pda, lysopa (20:2(11z,14z)/0:0), etc., they were ideal raw materials for the functional study of lipids and lipid-like molecules. MU, BR, and MQ were rich in glucotropaeolin, cichorioside K, and trehalose 6-phosphate, which are ideal raw materials for studying the function of organic oxygen compounds. In our comprehensive analysis, YS (0.8060), BR (0.7566), and ZD (0.7358) had the highest mean value of integrated membership function (Figure 7B), which indicated that YS, BR, and ZD had the most balanced and abundant nutrients, and their comprehensive nutritional quality was the best. These specimens are ideal raw materials for the development of medicinal functions. This study also found that YS, BR, ZD, MQ, GD, etc., with higher comprehensive evaluations were more distributed in the Qinghai–Tibet Plateau, while *C. sinensis* specimens around the Qinghai–Tibet Plateau ranked lower. This suggests that the climate, climatic conditions, and other natural environments in the Qinghai–Tibet Plateau have prompted *C. sinensis* to produce more diverse metabolites and improve their quality.

## 4. Discussion

The metabolites of *C. sinensis* are not only participants in their own physiological and biochemical processes, but they also play an important role in the individual’s resistance to biotic and abiotic stresses and have a variety of biological activities, which are beneficial to human health [24]. Although many studies have reported the composition and content of metabolites in *C. sinensis*, there is still a lack of comprehensive evaluation of the quality system of *C. sinensis* in the main producing areas of *C. sinensis* in China, which limits the edible value of *C. sinensis* as a functional food and the development of its medicinal value as a raw material for Chinese herbal medicine. To comprehensively evaluate the quality of *C. sinensis* from different producing areas, LC-MS/MS was used to qualitatively and quantitatively analyze the metabolites of *C. sinensis* from 15 producing areas. A total of 1718 metabolites were detected and annotated in all the samples, mainly carboxylic acids and derivatives, fatty acyls, organo-oxygen compounds, benzene and substituted derivatives, prenol lipids, glycerophospholipids, etc., which were the main nutrients of *C. sinensis* (Table S3), which was similar to the reported metabolites of *C. sinensis* in the literature [25,26]. Our multivariate statistical analysis showed that the metabolic spectrum characteristics of *C. sinensis* from different habitats were significantly different, which indicated that *C. sinensis* from different habitats had different metabolic accumulation patterns. The HCA analysis showed that the DAMs of *C. sinensis* from different habitats were mainly fatty acids and conjugates, fatty acid esters, glycerophosphocholines, carbohydrates, and carbohydrate conjugates. They also divided the 15 producing areas of *C. sinensis* into areas around the Qinghai–Tibet Plateau and in the Qinghai–Tibet Plateau, and the samples collected from the same producing area had a high similarity in metabolites, indicating that climatic conditions and a growth environment in the same range may be the main factors affecting the quality of *C. sinensis*. In general, the comprehensive quality of *C. sinensis* in the surrounding production region was relatively low, and the comprehensive quality of *C. sinensis* in the Qinghai–Tibet Plateau region was relatively high.

Fatty acids and conjugates, fatty acid esters, and glycerophosphocholines are lipids and lipid-like molecules, which not only have similar biological activities but also unique pharmacological effects. Glycerophosphocholines have been reported to inhibit adipocyte differentiation and pancreatic lipase activity at 100 μM, reducing fat accumulation and short-chain fatty acids in the mouse intestine [27]. The unsaturated fatty acids that it is rich in not only show anti-inflammatory effects by assisting in the scavenging of hydroxyl radicals but also promote the secretion of CAT, GSP-Px, and SOD, showing a strong antioxidant effect [28]. Fatty acids have a wide range of protective effects in cardiovascular diseases, digestive diseases, and autoimmune diseases, among which ω3FAs and docosahexaenoic acid (DHA) are considered to play an important role [29,30,31,32,33]. Carbohydrates and carbohydrate conjugates are one of the most common and abundant nutrients in edible fungi, providing energy to organisms through glycolysis and tricarboxylic acid (TCA) cycles, and they perform similar functions in *C. sinensis* [34]. In the anti-tumor activity test, D-mannitol is considered to enhance the phagocytosis of mouse macrophages and promote the release of immune active substances’ tumor necrosis factor-α (TNF-α) and interleukin-6 (IL-6) and improve the immune function of mice [35,36]. More animal experiments have also shown that AESP-II, composed of mannitol, glucuronic acid, rhamnose, galactose acid, and glucose, significantly increases the proliferation of T lymphocytes and B lymphocytes in a dose-dependent manner [37,38]. These studies have shown that *C. sinensis* provides a large amount of energy for organisms, and its secondary metabolites have a variety of medicinal functions. This study provides valuable data resources for the mining and exploration of the medicinal functions of *C. sinensis*.

Previous studies have shown that metabolites of *C. sinensis* grown in different environments change significantly [39,40]. This study found that the comprehensive quality of *C. sinensis* in the Qinghai–Tibet Plateau was higher than that around the Qinghai–Tibet Plateau, which may have been closely related to local climatic factors. The average altitude of BR, DQ, ZD, and YS in the western region was 4234 m, while the average altitude of XJ, SL, MU, and TZ in the eastern region was 3100 m. Even in the same producing areas, such as DQ, ZD, and BR, they were geographically close and had similar meteorological conditions. However, soil factors such as soil type and vegetation type could also lead to the accumulation of their metabolites (Table S1). According to the comprehensive ranking results of the quality of *C. sinensis* from different producing areas, the quality of *C. sinensis* in the western producing areas was generally higher than that in the eastern producing areas, indicating that the plateau climate in the west was more conducive to the accumulation of metabolites of *C. sinensis*. With the increase in altitude, this region shows a typical plateau climate such as low temperatures, low oxygen levels, large temperature differences between day and night, and reduced precipitations [41]. In this low-temperature environment, *C. sinensis* has a greater energy demand, accelerates carbon metabolism and carbohydrate synthesis, and accumulates more carbohydrates and their derivatives to adapt [35]. D-mannitol, as a high-quality reserve carbon source, is involved in multiple stress resistance processes of fungal cells [25]. At the same time, low temperature is also an important factor in inducing fatty acid accumulation [42,43], and fungal cell membranes resist temperature stress by forming interwoven membrane lipid structures [44]. Hypoxia leads to the inhibition of glycolysis and the TCA cycle. To obtain more energy, *C. sinensis* undergoes anaerobic respiration and accumulates more fatty acid compounds, which are involved in the fatty acid metabolism and synthesis [45,46]. At the same time, it leads to the accumulation of superoxide anions and oxygen-free radicals in *C. sinensis*. Lipids and lipid-like molecules rich in *C. sinensis* have a strong scavenging ability for these compounds. More animal experiments have shown that it has a strong antioxidant capacity [47]. Among the 18 biomarkers screened in this study, eight compounds, including 12-oxo-PDA, 1-Hexanol, and 12-methyltridecanoic acid, were derived from lipids and lipid-like molecules, and the content of these biomarkers was significantly different between the samples.

Meteorological conditions and environmental factors induce *C. sinensis* to produce different metabolites, resulting in differences in the quality of *C. sinensis* from different habitats. In this study, the comprehensive quality of *C. sinensis* from different producing areas was analyzed in detail. While explaining the price difference caused by different producing areas, it provided a scientific basis for consumers to choose *C. sinensis* for physical health care and valuable data for the exploration of more pharmacological effects of *C. sinensis*.

## 5. Conclusions

A total of 1718 metabolites were identified, which were divided into 14 superclasses and 130 classes in 15 producing areas. Among them, carboxylic acids and derivatives, fatty acids, organo-oxygen compounds, benzene and substituted derivatives, prenol lipids, and glycerophospholipids were the main components of *C. sinensis*. The HCA analysis of the top 50 metabolites of VIP value showed that the DAMs of *C. sinensis* from different habitats were mainly derived from fatty acids and their conjugates, fatty acid esters, carbohydrates and carbohydrate conjugates, and glycerophosphocholines. This study found and verified 18 biomarkers, which had a significant effect on the identification of the samples. The integrated membership function method was used to evaluate the nutritional quality of the 15 samples. The results showed that YS, BR, and ZD were nutritious, excellent raw materials for the development of functional foods, and good choices for health care. YS, ZD, and DQ were rich in 12-methyltridecanoic acid, 12-oxo-pda, lysopa (20:2 (11z,14z)/0:0), and so on, which made them ideal raw materials for the functional study of lipids and lipid-like molecules. MU, BR, and MQ were rich in glucotropaeolin, cichorioside K, and trehalose 6-Phosphate, which made them ideal materials for studying the function of organic oxygen compounds. In summary, this study provided a basis for the edible value, medicinal value, and grading identification of *C. sinensis*.

## Data Availability

Data are contained within the article and Supplementary Materials.

## Supplementary Materials

The following supporting information can be downloaded at https://www.mdpi.com/article/10.3390/jof10050356/s1, Figure S1: Total ion chromatograms of samples in positive- and negative-ion modes for untargeted metabolomics methods; Figure S2: Single factor analysis of top 50 DAMs. The capital letters in the figure indicate significant differences in metabolite abundance at the *p* < 0.05 level; Table S1: Meteorological data of sampling sites; Table S2: Correlation analysis between 18 biomarkers and sampling sites; Table S3: Details of the compounds obtained by KEGG annotation; Table S4: Classification information for all the compounds (those which cannot be classified have been deleted); Table S5: Enrichment analysis of KEGG pathways; Table S6: Comprehensive membership function values and ranking of 15 groups samples.
