# Supplementary figures and images for: Evaluation of Cordyceps sinensis Quality in 15 Production Areas Using Metabolomics and the Membership Function Method
Source: J Fungi (Basel). 2024 May 16;10(5):356. doi: 10.3390/jof10050356 (PMC11122220; doi:10.3390/jof10050356)

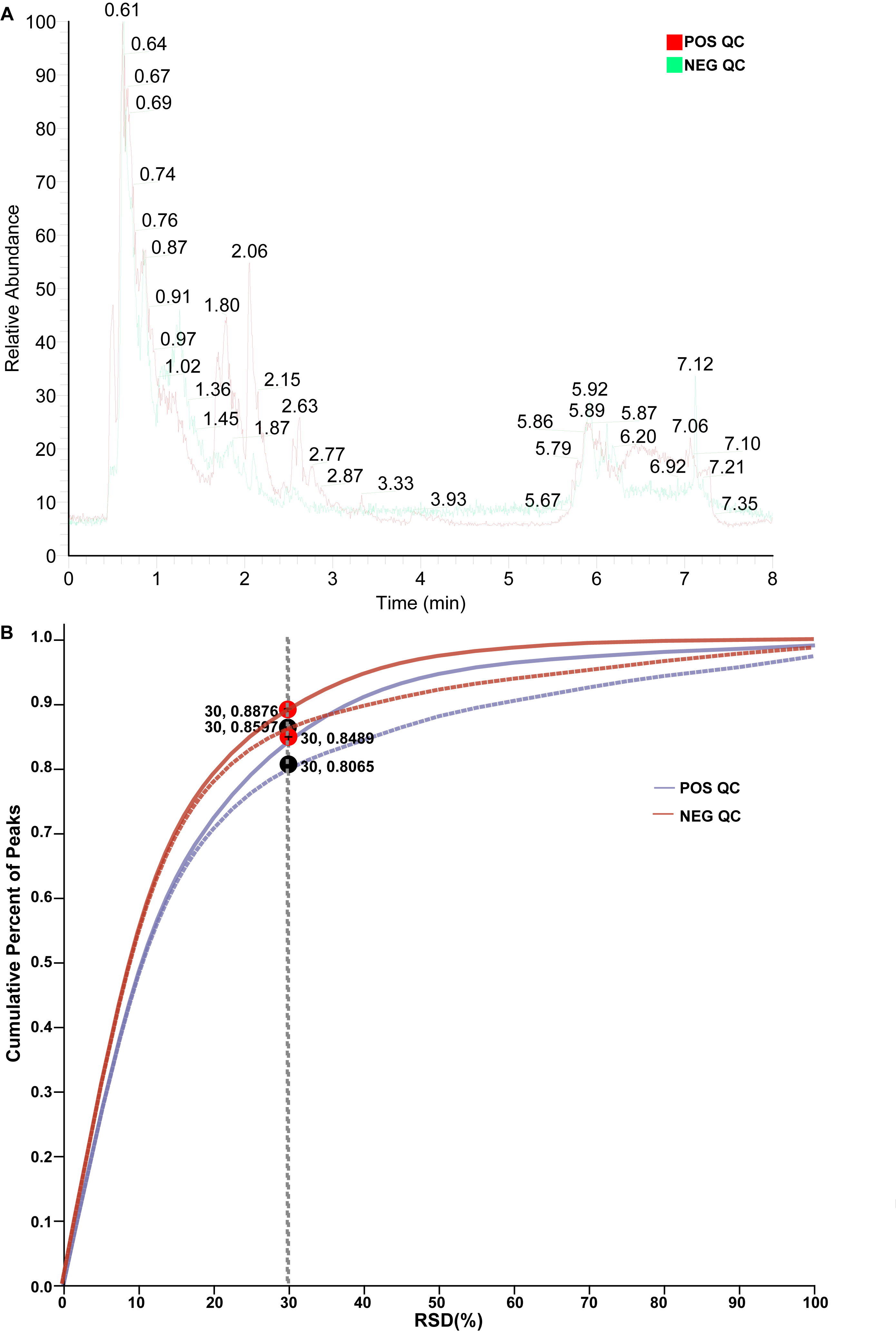

Supplement: Supplementary file 1 [file jof-10-00356-s001.zip › Figure S1.Total ion chromatograms of samples in positive- and negative-ion modes for untargeted metabo-lomics methods.png]

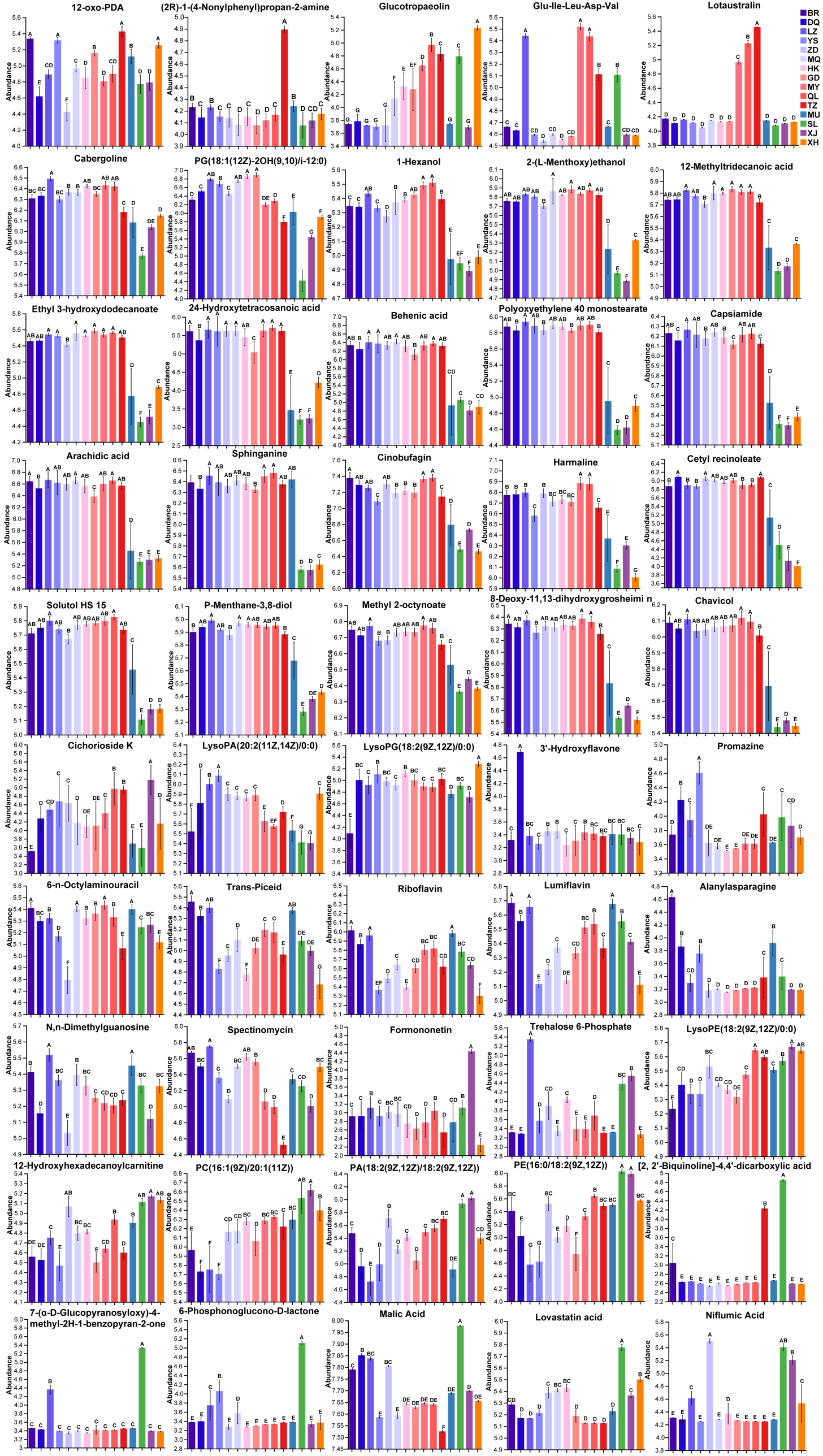

Supplement: Supplementary file 1 [file jof-10-00356-s001.zip › Figure S2.Single factor analysis of top 50 DAMs. The capital letters in the figure indicate significant differ-ences in metabolite abundance at the p 0.05 level.png]
